# Sera from Patients with Malignant Pleural Mesothelioma Tested Positive for IgG Antibodies against SV40 Large T Antigen: The Viral Oncoprotein

**DOI:** 10.1155/2022/7249912

**Published:** 2022-07-15

**Authors:** Elisa Mazzoni, Ilaria Bononi, John Charles Rotondo, Chiara Mazziotta, Roberta Libener, Roberto Guaschino, Roberta Gafà, Giovanni Lanza, Fernanda Martini, Mauro Tognon

**Affiliations:** ^1^Department of Chemical, Pharmaceutical and Agricultural Sciences—DOCPAS, University of Ferrara, Ferrara 44121, Italy; ^2^Department of Translational Medicine and for Romagna, University of Ferrara, Ferrara 44121, Italy; ^3^Department of Medical Sciences, Section of Experimental Medicine, School of Medicine, University of Ferrara, Ferrara 44121, Italy; ^4^Mesothelioma BioBank, Pathology Unit, City Hospital, Alessandria, Italy; ^5^Transfusion Medicine, City Hospital, Alessandria, Italy; ^6^Section of Pathology, Department of Translational Medicine, School of Medicine, University of Ferrara, Ferrara, Italy; ^7^Laboratory for Technologies of Advanced Therapies, Department of Medical Sciences, University of Ferrara, Ferrara 44121, Italy

## Abstract

Malignant pleural mesothelioma (MPM), a fatal tumor, is mainly linked to the asbestos exposure. It has been reported that together with the inhalation of asbestos fibers, other factors are involved in the MPM onset, including simian virus 40 (SV40). SV40, a polyomavirus with oncogenic potential, induces (i) in vitro the mesenchymal cell transformation, whereas (ii) in vivo the MPM onset in experimental animals. The association between MPM and SV40 in humans remains to be elucidated. Sera (n = 415) from MPM-affected patients (MPM cohort 1; n = 152) and healthy subjects (HSs, n = 263) were investigated for their immunoglobulin G (IgG) against simian virus 40 large tumor antigen (Tag), which is the transforming protein. Sera were investigated with an indirect enzyme-linked immunosorbent assay (ELISA) using two synthetic peptides from SV40 Tag protein. SV40 Tag protein was evaluated by immunohistochemical (IHC) staining on MPM samples (MPM cohort 2; n = 20). Formalin-fixed and paraffin-embedded (FFPE) samples were obtained from MPM patients unrelated to MPM serum donors. The proportion of sera, from MPM patients, showing antibodies against SV40 Tag (34%) was significantly higher compared to HSs (20%) (odds ratio 2.049, CI 95% 1.32–3.224; *p*=0.0026). Immunohistochemical staining (IHS) assays showed SV40 Tag expression in 8/20, 40% of MPM specimens. These results indicate that SV40 is linked to a large fraction of MPM. It is worth noting that the prevalence of SV40 Tag antibodies detected in sera from cohort 1 of MPM patients is similar to the prevalence of SV40 Tag found to be expressed in FFPE tissues from MPM cohort 2.

## 1. Introduction

Malignant pleural mesothelioma (MPM) is a highly aggressive tumor arising from the mesothelium of the pleural surface [1]. MPM is responsible for 4% of cancer deaths [2]. This malignancy is considered a rare cancer, but in recent years, MPM cases have increased significantly. Indeed, MPM accounts approximately 40,000 deaths/year worldwide [3]. MPM is linked to asbestos, which was employed as different tumorigenic natural mineral fibers [4]. The incidence of MPM is variable among different countries, whereas it has been estimated that this cancer will increase its incidence worldwide in the subsequent 20 years [4, 5]. Currently, MPM causes approximately 5,000 and 3,000 deaths/year in western Europe and the USA, respectively [4,5].

The MPM onset is predominant in males, whereas 80% of cases result from the asbestos exposure in the workplace. However, 20% of MPM arises in patients not exposed to asbestos fibers [6]. Many investigations support a clear link between asbestos fiber exposure and the subsequent MPM onset [7]. Indeed, asbestos fibers have been established to be the cause of the MPM onset/progression, which may occur up to 50 years after the asbestos exposure [4,5]. In this context, it is worth recalling that asbestos is a general term employed for regulatory purposes to identify six out of about 400 mineral fibers commercially distributed. It has been estimated that several millions of individuals have been contaminated with asbestos fibers worldwide [8]. MPM incidence is increasing because this carcinogenic mineral was massively employed for decades [3]. Many reports and clinical evidence have confirmed the asbestos carcinogenic properties [4,5]. It has been reported that MPM arises in ex-exposed asbestos workers with a prevalence, depending on the studies, in the range of 1–10% [1]. However, 20% of MPM arises in patients not exposed to asbestos fibers [6].

MPM is becoming a significant health problem due to its increasing incidence [9] and the absence of efficient therapies/treatments. Among MPM peculiarities, the poor prognosis and median survival, less than 1 year from the time of diagnosis, can be accounted [9]. In addition, in the early step of the MPM onset, the tumor appears asymptomatic or the clinical symptoms are not specific. Consequently, MPM is often recognized at advanced stages for an appropriate treatment [10]. Altogether, these considerations indicate the need of novel strategies for diagnosis, prognostication, and effective treatments.

Together with asbestos fibers, there is a need to verify whether additional factors, such as the presence of asbestos fibers in the environment, other mineral fibers, ionizing radiation, or infections by oncogenic viruses, are associated with the onset of this fatal cancer. Together with the environmental factors, the genetic background of the host seems to predispose to the MPM onset [3,4].

Among oncogenic viruses [11], the transforming polyomavirus simian virus 40 (SV40) was associated with MPM, as an additional risk factor. Indeed, SV40 was proposed as a potential cofactor in the MPM onset/progression [12,13].

SV40 sequences and expression of its Tag viral oncoprotein were revealed by several groups in MPM tumor tissues, whereas other investigators reported negative data [12,14–19]. Indeed, SV40 DNA has been detected in mesotheliomas and other human tumors of different histotypes. [17,20,21] In addition, it has been reported that mesothelioma cells/MPM tissues tested positive for SV40 Tag expression [22–25].

SV40-contaminated antipolio vaccines, which were administered in different amounts in distinct countries, are considered the main source of SV40 infection in humans [17, 26]. However, new data suggest that SV40 circulates at present in some populations independently from contaminated vaccines [27].

It has been published that SV40 viral oncogenes act together with asbestos fibers in the carcinogenetic process [28]. Indeed, SV40 oncogenes [29] synergistically contribute in vivo, with the asbestos fibers, to the MPM onset [30]. The SV40 transforming activity is due to the Tag expression, which binds and inactivates the products of tumor suppressor genes pRB and p53 [17,20,21]. It is worth recalling that SV40 Tag owns a high powerful transformation activity [17].

It has been reported that workers ex-exposed to asbestos fibers and SV40-infected have a risk 12.6 times higher to develop MPM compared to subjects without the two risk factors or with only one [24]. The association or interaction between SV40 and MPM in humans is poorly known and remains to be elucidated.

This study was undertaken to investigate the putative link between MPM (cohort 1) and SV40 viral oncoprotein. To this purpose, an indirect ELISA test with synthetic peptides mimicking Tag epitopes was employed to capture serum IgG antibodies from MPM patients and healthy subjects (HSs).

In addition, Tag expression was tested in MPM biopsies by immunohistochemical (IHC) technique. These MPM samples (cohort 2), randomly chosen from our pathology archive, were unrelated to the serum donors MPM patients.

## 2. Materials and Methods

### 2.1. Samples, MPM Patients, and Healthy Subjects

Serum samples (n = 415) of our collection were from MPM (n = 152) and healthy subjects (HSs, n = 263) [31,32]. Different cohorts were homogeneously clustered according to age and gender. Inclusion criterion was MPM diagnosis for MPM patients. The control group were recruited retrospectively from our serum collection. Sera of the control group were obtained from healthy adult subjects (HSs) (>18 yrs), that is, without neoplasia, autoimmunity disease, and inflammatory status. HSs as blood donors were in normal conditions, as reported in the hospital records. HSs were admitted to the Clinical Laboratory Analysis of our University Hospital (Sant'Anna Hospital, Ferrara, Italy) for routine blood analyses during a general check-up. This study was approved by the Ethical Committee (EC) of the Province of Ferrara, Italy (protocol number: 151078). The study was conducted in accordance with the principles of scientific research set out in the Helsinki Declaration. The mean age of MPM patients and HSs was 68 years. The gender proportion was 68% and 62% for MPM patients and HSs, respectively.

### 2.2. Indirect Enzyme-Linked Immunosorbent Assays

Indirect enzyme-linked immunosorbent assays (ELISAs) were employed to analyze serum samples from MPM patients and HSs. Serum IgG antibodies against SV40 Tag were investigated with ELISA. The immunological test employed herein has been recently published [31]. Briefly, in indirect ELISAs, serum IgG antibodies against SV40 Tag were detected using mimotopes as synthetic peptides known as SV40 Tag A and D [31]. The two specific SV40 Tag peptides were selected by computer-assisted analyses. SV40 Tag mimotopes (A and D) did not cross-react with BKPyV and JCPyV hyperimmune sera, employed as negative control [31]. Amino acid sequences of the two Tag A and D peptides are from a.a. residues 669–689 (21 a.a.) and from 659 to 682 (24 a.a.), respectively, as reported [31]. These peptides/mimotopes were selected from specific Tag domains, which were exposed to the polypeptide surface, as reported before. Serum samples were diluted in a low cross-buffer at a 1 : 20 (Candor Bioscience). This high concentration is required to increase the amount of antibodies against the few epitopes present in short synthetic peptides. ELISA was carried out in several phases/steps: (i) peptide coating, (ii) peptide blocking, (iii) primary and (iv) secondary antibody additions, and (v) dye treatment and spectrophotometric reading at a wavelength (*λ*) of 405 nm (Thermo Electron Corp., model Multiskan EX, Finland). The cut-off was determined as reported. The apparatus “Wellwash 4 Mk 2” (Thermo Electron Corp, Vantaa, Finland) was used in ELISA to remove the solutions/sera and rinse the plates. The Wellwash 4 Mk 2 is a semiautomatic microplate washer for 96-well plate, comprising pump and washer units.

- Peptide coating: plates and blocking. Ninety-six well flat-bottom wells (Nunc-immuno plate PolySorp, CelBio, Milan, Italy) were coated with 5 µg of the selected peptide for each well, which were diluted in 100 µL of coating buffer (Cat number: 010CNB121125, Candor Bioscience, Weissenberg, Germany). The plates were left at 4°C for 16 hours to allow the peptide to completely cover the bottom well. To eliminate uncoated peptide, washing buffer was used to rinse the plates three times (Candor Bioscience, Weissenberg, Germany)
- Blocking phase was made with 200 µL/well of the blocking solution (Cat number: 010CNB110500, Candor Bioscience, Weissenberg, Germany) at 37°C for 90 minutes to saturate the wells. To remove the blocking solution, washing buffer was used three times to rinse the plates (Cat number 010CNB145500 Candor Bioscience, Weissenberg, Germany).
- Primary antibody. 96 plastic wells were covered with 100 µL containing the following sera: positive control represented by (a), positive control represented by (ia), immune rabbit serum containing anti-SV40 Tag antibodies [31], and (ii-a) human sera found to be SV40 Tag-positive with neutralizing activities in a previous study [31] were employed as additional positive controls in all indirect ELISA carried out with SV40. Control sera gave optical density readings of 0.25–0.72. In addition (b) negative controls were employed and these sera are represented by (i-b) rabbit hyperimmune sera against BKPyV and JCPyV obtained performed previously [31, 33] (ii-b) three human serum samples found to be SV40 negative neutralizing activities in a previous study [31] in addition (iii-b) a human negative peptide, the neuropeptide S (hNPS) with the a.a. sequence SFRNGVGTGMKKTSFQRAKS, was employed as a control. This human neuropeptide is non-liked to SV40 [31,33]. Peptides were synthesized with standard procedures and purchased from the University of Ferrara firm, the UFPeptides s.r.l., Ferrara, Italy. The OD value usually in the range of 0.050–0.080, consistent with the OD of SV40-negative sera
- Secondary antibody. After 90 minutes of incubation, a new triple rinsing cycle was repeated as described above. Then, in each well, the secondary human antibody solution was added. The secondary antibody is a goat anti-human or anti-rabbit immunoglobulin G (IgG) heavy (H)- and light (L)-chain-specific peroxidase conjugate (Calbiochem-Merck, Darmstadt, Germany) diluted 1 : 10,000 in low cross-buffer (Cat number 010CNB100500 Candor Bioscience, Weissenberg, Germany). The reaction mixture was incubated at room temperature for 90 minutes. Then, the plates were washed three times with the washer buffer and treated with 100 µL of 2,2 = -azino-bis 3-ethylbenzthiazoline-6-sulfonic acid solution, ABTS (Cat number 30931-67-0, Sigma-Aldrich, Milan, Italy). The colorimetric process was stopped after 45 minutes with 100 L of 0.1 M citric acid.
- Plate spectrophotometric reading. The spectrophotometer (Model Multiskan EX, Thermo Electron Corp) was used to read the plate at a wavelength (*λ*) of 405 nm. Based on the presence of specific antibodies binding to SV40 synthetic peptides/epitopes/mimotopes, immunocomplexes displayed a different color intensity in wells, which was revealed by a distinct optical density (OD). The cut-off was determined in each assay by the OD mean reading of the three negative control sera, added to the standard deviation (SD) three times (+3 SD) [31,33]. By visualizing the ranking net OD individual value for each peptide, the three SV40-negative controls were chosen from those below the cut-off value, defined by second-degree polynomial regression [31]. Graphical data show an inflection point for peptide A and peptide D at 0.19–0.18, respectively. Immune sera were considered to be SV40 Tag-positive when reacting to both peptides A and D, in three replica experiments carried out by independent operators with no data variability.

### 2.3. Immunohistochemical (IHC) Analysis

Immunohistochemical (IHC) analysis was performed on formalin-fixed and paraffin-embedded (FFPE) MPM specimens (n = 20). SV40 Tag expression protein was evaluated in randomly chosen MPM specimens from the pathology archive. IHC staining was carried out by using the Multimeric Detection Kit (Universal DAB Detection Kit Ultraview, Roche Tissue Diagnostics [CH]), on a BenchMark XT immunostainer (Roche T. D.). [34] FFPE slices, 4µ thick, were allowed to react to the mouse monoclonal antibody against the SV40 Tag (Pab 101, Santa Cruz Biotechnology, Santa Cruz, CA) (dilution, 1 : 50) [35]. Pathologists of our working group evaluated the staining intensity and dispersion. Staining was graded as negative (no staining) or weak/moderate/strong intensity, as reported before in IHC analyses for SV40 Tag [36,37].

### 2.4. Statistical Analysis

Sera from MPM patients and HSs were analyzed to compare the prevalence of SV40 Tag-positive sera. Statistical analyses were performed with chi-square with Yates' correction test. The serologic profile of the reactivity to SV40 Tag mimotopes was statistically analyzed using the Mann–Whitney test. Prism 7.0 (GraphPad software, San Diego, CA) was used for computational analyses. For all tests, p was considered to be statistically significant when *p* < 0.05 [31].

## 3. Results

### 3.1. Prevalence of IgG Antibodies against SV40 Tag Detected in Sera from Malignant Pleural Mesothelioma-Affected Patients

**Figure 1:**
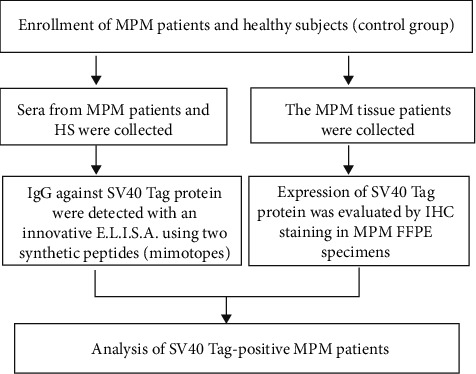
The general research design and flow of the observational study.

This study was performed to verify the putative link between MPM and SV40 Tag (Figure 1). Sera of malignant pleural mesothelioma-affected patients and healthy subjects were detected in sera collected in 2010 and 2019. Serum samples (n = 415) from our collection were obtained from MPM (n = 152) and healthy subjects (HSs, n = 263). Sera from MPM patients and HSs, employed as control, were investigated by indirect ELISAs using SV40 Tag mimotopes. Specifically, samples were tested for the presence SV40 Tag antibodies. This investigation used a new specific indirect ELISA with mimotopes/antigens of SV40 Tag, named peptides A and D [31].

**Figure 2:**
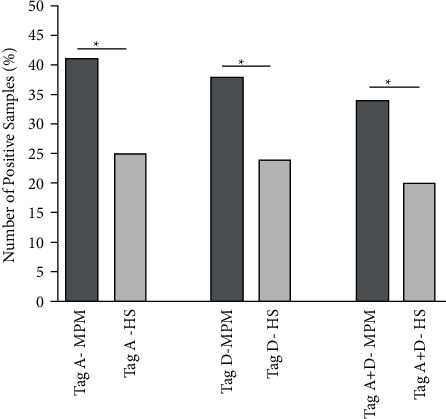
Prevalence of IgG antibodies reacting to SV40 Tag mimotopes in sera from MPM patients and healthy subjects. Sera from MPM patients and HS were analyzed to compare the prevalence of SV40 Tag-positive sera. The prevalence of IgG antibodies was expressed with number of positive samples (%). Immunological data are from MPM and HS with the same mean age and gender. The different prevalence of SV40 Tag antibodies (Tag A + D) in the MPM cohort is statistically significant compared to HS (odds ratio 2.049, CI 95%: 1.32–3.224; ^*∗*^*p*=0.0026). Statistical analysis was performed by the *χ*2 test with Yates' correction.

In the first step of our immunological assay, MPM sera diluted to 1/20 were analyzed for their reactivity to mimotope A, as reported before in other studies with different polyomavirus mimotopes [31,33]. MPM sera reacted to the SV40 Tag A mimotope with a prevalence of 41% (n = 62/152), whereas HSs reached 25% (n = 67/263) (Table 1, Figure 2).

Then, the indirect ELISA with the mimotope D was used to capture serum IgGs. It this assay, MPM patients and HSs sera reached a prevalence of 37% (57/152) and 24% (64/263), respectively (Table 1).

In our analyses, only those MPM and HS samples reacting to both Tag mimotopes A and D were considered positive.

The prevalence of MPM and HS samples reacting to both A and D mimotopes was 34% (51/152) and 20% (52/263), respectively (Table 1, Figure 2), being the different prevalence of the two cohorts, cases vs control, statistically significant (odds ratio 2.049, CI 95%: 1.32–3.22; *p*=0.0026) (Table 1, Figure 2).

### 3.2. Serological Profiles

**Figure 3:**
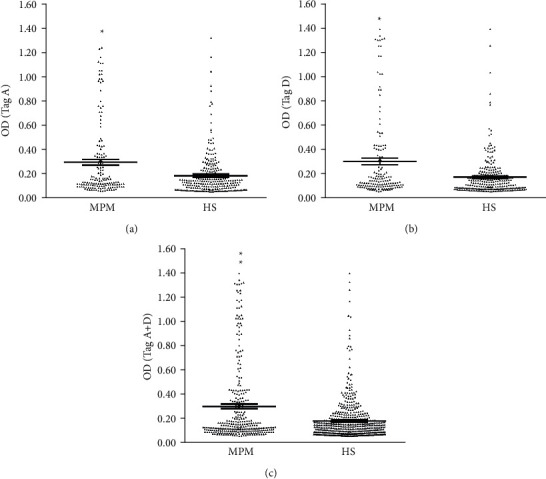
Serologic profiles of antibody reactivity to SV40 Tag mimotope A (a), mimotope D (b), and mimotopes A + D (c) quantified in MPM and HS sera. Sera from MPM patients and HS were analyzed to compare the prevalence of SV40 Tag-positive sera. Immunological data are from MPM and HS with the same mean age and gender. The mean OD value of serum antibodies against SV40 Tag mimotopes A, D, and A + D was higher in MPM patients vs HS (^*∗*^*p* < 0.0001; ^*∗∗*^*p* < 0.0001).

Serological profiles of antibodies, which reacted to SV40 Tag synthetic peptides, are displayed in the scatter dot plotting. The single plot indicates the dispersion of each sample OD value to a mean level (ML). The ML is represented with a long horizontal bar within the scatter, where the standard error of the mean (SEM) is indicated with a short horizontal bar for each group (Figure 3).

The results were elaborated by means of the Mann–Whitney assay and displayed as OD mean, CI 95%. The mean OD value of serum SV40 Tag antibodies was higher in MPM (OD = 0.30, 95% CI: 0.26–0.34) vs HSs (OD = 0.18, CI 95%: 0.17–0.19; ^*∗∗*^*p* < 0.0001). The control sample that tested SV40-positive was the hyperimmune anti-SV40 Tag rabbit serum. This serum reacted to both Tag A and D peptides, with OD of 2.8 and 2.3, respectively [31]. Positive controls represented by human sera that tested positive for SV40 Tag epitopes showed ODs with a mean value of 0.30–1.50 [31]. Negative controls were (i) rabbit hyperimmune sera against BKPyV and JCPyV and (ii) three human serum samples found to be SV40 negative in terms of neutralizing activities as reported in a previous study [32]. These sera did not react against SV40 Tag A and D mimotopes showing OD range value of 0.07–0.012 and 0.015–0.016, respectively. These results are in agreement with data obtained before [31]. The neuropeptide hNPS, which is SV40-unrelated, was employed as human negative peptide/mimotope. HNPS did not react with all sera, with ODs in the range of 0.052–0.085 [31].

Interestingly, serum MPM antibodies had a significant higher OD for the mimotope A (OD = 0.30, CI 95% = 0.25–0.35) vs HSs (OD = 0.19 CI 95% = 0.16–0.21; ^*∗*^*p* < 0.001). Similar data were obtained for the D mimotope in MPM patients (OD = 0.30, CI 95% = 0.25–0.36) vs HSs (OD = 0.17, CI 95% = 0.15–0.19; ^*∗*^*p* < 0.001). In agreement with the single OD values, the OD value for A + D mimotopes was higher in MPM (OD = 0.30, CI 95% = 0.26–0.34) vs HSs (OD = 0.18, CI 95% = 0.17–0.20; ^*∗∗*^*p* < 0.0001), Figure 3.

### 3.3. SV40 Tag Expression in MPM FFPE Specimens

**Figure 4:**
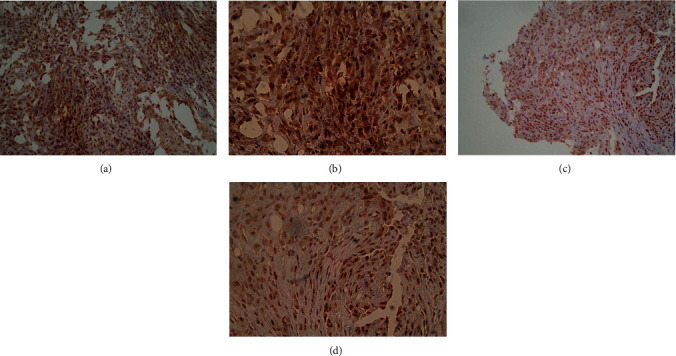
Histological staining of MPM slices with the monoclonal antibody Pab 101 against the SV40 Tag oncoprotein. (a) and (b). These panels show the epithelioid component of a biphasic mesothelioma sample with all malignant cells found to be SV40 Tag-positive; (a) magnification 200×; (b) magnification 400×. (c) and (d). These panels show the same epithelioid mesothelioma sample. All malignant cells tested SV40 Tag-positive; (c) magnification 200×; (d) magnification 400×.

**Figure 5:**
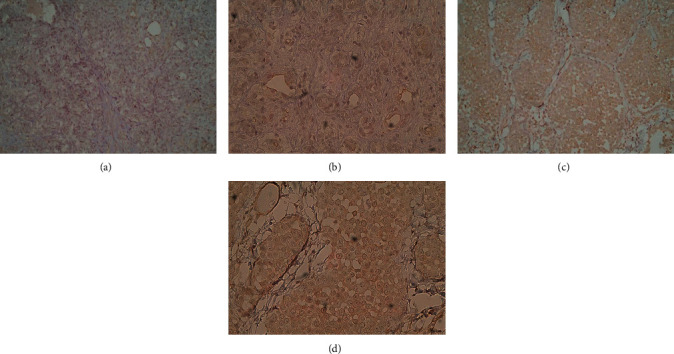
Histological staining of MPM slices with the monoclonal antibody Pab 101 against the SV40 Tag oncoprotein. (a) to (d) These panels show two epithelioid MPM samples found to be SV40 Tag-negative; (a and c) magnification 200×; (b and d) magnification 400×.

To investigate the SV40 Tag protein expression, the IHC method was used in FFPE tissues (n = 20) from MPM patients. Considering that 34% of MPM sera tested SV40 Tag-positive, we reasoned that randomly chosen MPM FFPE specimens, from unrelated MPM patients, could express SV40 Tag. To this purpose, IHC with a specific mab against SV40 Tag was carried out in slices from MPM FFPE (n = 20) samples taken from our pathology archive. The IHC analysis showed that MPM specimens, 8 out of 20 (40%), tested SV40 Tag-positive. Specifically, in these 8 MPM specimens, the SV40 Tag was detected with diffuse or dot-like nuclear localization, with a variable range of weak/moderate/strong signals in different samples (Figures 4(a) and 4(b)). In other MPM specimens (n = 10), SV40 Tag expression was not detected (Figures 5(a) and 5(b)), whereas not assessable in one sample.

## 4. Discussion

Many different data were reported in the literature on the association of SV40 with human tumors and its circulation in human populations [15]. In previous reports, the link between MPM and SV40 was assessed by employing distinct methods, such as PCRs, southern and in situ hybridizations, western blot, immunohistochemistry, and immunological tests [1,17,21,24,38]. Other studies did not support the association [12,14–17], including the lack of specific SV40 Tag mRNA in MPM tissue samples from patients contaminated by asbestos and administered with antipolio vaccines tested SV40-positive [19].

Some of these discrepancies could be related to the DNA sequence similarity of different polyomaviruses, which share approximately 70% of their DNA sequences. Furthermore, early immunological assays, which were employed as a viral antigen, the recombinant VP1 or VLPs, always showed cross-reactivity among the three polyomaviruses, thus affecting the specificity of immunological data [17,39].

This investigation was addressed to verify the association of MPM with the SV40 Tag. To this purpose, MPM sera were tested for SV40 Tag antibodies, and indirect ELISAs with SV40 Tag peptides were employed. Data indicated that MPM sera tested SV40 Tag-positive with a prevalence higher (34%) than HSs (20%), the difference being significant. In addition, the serologic profile results indicate that OD values obtained for MPM sera are significantly higher than HSs. Altogether, these data support the link between MPM and SV40 Tag, in a subset of patients (34%).

Of note, the detection of SV40 Tag antibodies in HSs, although at a lower prevalence (20%), supports the hypothesis that this or a very closer polyomavirus circulates in human populations.

In a previous study, we showed that sera from MPM patients react to SV40 viral proteins 1-3 (VPs) at a significantly higher prevalence than HSs, with the same median age and gender [32].

Our indirect ELISA is a reliable and specific approach to discriminate SV40 Tag-negative from SV40 Tag-positive MPM patients and HSs. In earlier investigations, employing the same immunological approach, we detected serum antibodies against SV40 Tag in HSs in the range of 18%–19%. This prevalence did not differ substantially in the HSs of different ages [31,33]. The expression of SV40 Tag detected in MPM FFPE specimens by immunohistochemistry staining showed a diffuse or dot-like nuclear localization of the viral oncoprotein in 8 out of 20 (40%) MPM FFPE tissues. Of note, the 40% prevalence of MPM tissues, which tested SV40 Tag-positive, is similar to that (34%) of SV40 Tag-positive sera from MPM patients.

The two MPM cohorts, cohorts 1 and 2, are independent of each other. Indeed, MPM sera were anonymously collected. Consequently, it was impossible to IHC-analyze the MPM tissues of the same patients.

Herein, a new immunological assay with SV40 Tag peptides detected and quantified SV40 Tag antibodies in sera from MPM patients. The indirect ELISA in our experimental conditions appears specific for SV40 antigens. In fact, it allowed us to circumvent the nonspecific reactivity among homologous polyomaviruses. In agreement with the immunological data, the expression of SV40 Tag, the viral oncoprotein, was revealed by immunohistochemical (IHC) staining in randomly chosen MPM specimens.

It would be possible that the activity of SV40 Tag is exerted together with the asbestos fibers during the MPM development, in a manner similar to that demonstrated in vitro in transforming normal mesothelial cells. [40] Some elderly individuals among workers ex-exposed to asbestos fibers, during the immune-senescence phase, could be unable to control the oncogenic activities of both asbestos fibers and SV40 Tag. This scenario may occur in genetically predisposed subjects. This hypothesis is in agreement with the multistep mechanism of oncogenesis, which may occur during the tumor onset/development. Since SV40 seems to act as a cofactor in human MPM, novel therapies and preventive approaches should be employed in clinical applications. Indeed, the two distinct SV40 Tag-positive and SV40 Tag-negative MPM patients could be treated differently.

It should be recalled that the detection of SV40 Tag antibodies and Tag expression in MPM samples does not constitute a proof that this oncogenic polyomavirus is the infectious agent causing the MPM onset.

## 5. Conclusion

In summary, this study reports SV40 Tag antibodies in MPM sera with a significant higher prevalence than HSs. In agreement, the expression of SV40 Tag, which is its main oncoprotein, was shown by IHC staining in MPM specimens randomly chosen from our archive. The detection of SV40 antibodies and Tag expression in MPM samples indicate an association, but do not represent a proof that SV40 is responsible of the tumor onset. It would be possible that some individuals are unable to control the oncogenic activities of both asbestos fibers and SV40 Tag. We may speculate that after the SV40 infection, subjects/workers exposed to asbestos fibers are more sensitive/prone to develop MPM. Since SV40 seems to act as a cofactor in human MPM, new therapeutic/preventive strategies could be employed in clinical applications. In addition, novel immunological assays could represent a tool to detect the SV40 Tag antibodies response in individuals ex-exposed to asbestos fibers.

## Figures and Tables

**Table 1:** Prevalence of IgG antibodies reacting to SV40 Tag mimotopes in sera from MPM patients and healthy subjects.

Serum	Number of patients/individuals	Age range years (mean)	Male %	Number of positive samples (%)		
				Tag A	Tag D	Tag (A + D)
MPM	152	37–89 (68)	68	62 (41)	57 (38)	51(34)^*∗*^
HS	263	49–100 (68)	62	67 (25)	64 (24)	52 (20)

Abbreviation: MPM, malignant pleural mesothelioma; HS, healthy subjects; IgG, immunoglobulin G; SV40, simian virus 40; Tag, Large T antigen; Tag A and Tag D, synthetic peptides/mimotopes employed in ELISAs to detect SV40 Tag antibodies. Different cohorts were homogeneously clustered according to age and gender. The mean age of MPM patients and HS was 68 years. The different prevalence of SV40 Tag antibodies in the MPM cohort is statistically significant compared to HS (odds ratio 2.049, CI 95%: 1.32-3.224; ^*∗*^*P*=0.0026). Statistical analysis was performed by the *χ*2 test with Yates' correction. For all tests, *p* was considered to be statistically significant when *P* < 0.05. All computational analyses were performed with Prism 6.0 (GraphPad software, San Diego, CA).

## Data Availability

The data used to support the findings of this study are included within the article.
